# Primary Myeloid Sarcoma of the Breast Mimicking Carcinoma: Aberrant Expression of PAX5 and KMT2A::MLLT10 Fusion

**DOI:** 10.7759/cureus.111135

**Published:** 2026-06-19

**Authors:** Chaimae Bekhakh, Anass Haloui, Nassira Karich, Amal Bennani

**Affiliations:** 1 Pathology Department, Mohammed VI University Hospital, Faculty of Medicine and Pharmacy of Oujda, Mohammed First University of Oujda, Oujda, MAR

**Keywords:** acute myeloid leukemia, breast involvement, extramedullary leukemia, myeloid sarcoma, primary breast tumor

## Abstract

Myeloid sarcoma (MS) is a rare extramedullary tumor characterized by the proliferation of immature myeloid precursor cells. It most commonly occurs in association with acute myeloid leukemia, either at initial diagnosis or during disease progression. Breast involvement is exceedingly rare and represents a significant diagnostic challenge, as it can closely mimic more common breast malignancies, particularly carcinoma or lymphoma, frequently leading to misdiagnosis. We report the case of a 39-year-old woman who presented with two bilateral breast masses, initially suspected to be malignant based on clinical and radiological findings. Histopathological examination revealed a poorly differentiated malignant neoplasm composed of immature cells with eosinophilic cytoplasm and prominent nucleoli, diffusely infiltrating the mammary parenchyma. Immunohistochemical analysis demonstrated strong positivity for leukocyte common antigen (LCA), myeloperoxidase (MPO), and CD68, with focal expression of CD34 and CD117, supporting the diagnosis of primary bilateral breast MS; notably, aberrant expression of PAX5, a marker usually associated with B-cell lineage, constituted a potential diagnostic pitfall. Molecular analysis further identified a KMT2A::MLLT10 fusion, a rearrangement associated with monocytic differentiation and extramedullary involvement. Bone marrow biopsy showed no evidence of neoplastic infiltration. The patient was treated with combined chemotherapy and radiotherapy. This case highlights the diagnostic challenges posed by bilateral breast MS and underscores the critical role of histopathology, immunohistochemistry, and molecular studies in establishing an accurate diagnosis and guiding appropriate management.

## Introduction

Myeloid sarcoma (MS) was initially reported by Burns in 1811 and was later recognized as a manifestation of myeloid neoplasms by Dock in 1893 [1]. According to the most recent World Health Organization (WHO) Classification of Tumours of Haematopoietic and Lymphoid Tissues, MS is defined as a mass-forming extramedullary proliferation of myeloid blasts causing tissue destruction and arising at any anatomical location outside the bone marrow compartment [2,3].

MS may present in various clinical settings, including as an initial manifestation preceding acute myeloid leukemia (AML), concomitant with AML at diagnosis, as a manifestation of disease relapse, or as a result of blastic transformation of underlying myelodysplastic syndrome, myelodysplastic/myeloproliferative neoplasm, or myeloproliferative neoplasm. Consequently, the diagnosis of MS is considered biologically and clinically equivalent to AML and requires similar therapeutic management [4,5]. Large case series have shown that approximately one-quarter of MS cases occur in the absence of concurrent bone marrow involvement or previously diagnosed myeloid neoplasia [6].

MS can affect a wide range of extramedullary sites, most frequently involving the skin, lymph nodes, gastrointestinal tract, bone, and central nervous system. In contrast, primary involvement of the breast is exceptionally uncommon, with only isolated cases described in the literature to date [6].

## Case presentation

A 39-year-old woman with no family history of breast cancer presented with a five-month history of a rapidly growing mass in the right breast, which she had discovered spontaneously. Over the following weeks, she developed a second mass in the left breast, resulting in bilateral involvement. The masses were associated with no overlying skin changes, nipple discharge, or constitutional symptoms such as fever, night sweats, or weight loss. She had no relevant past medical or surgical history, no known comorbidities, and was not taking any regular medication. She was premenopausal with a regular menstrual cycle.

Clinical examination revealed a large firm mass measuring approximately 4.3 × 2.8 cm in the upper inner quadrant of the right breast. In addition, a smaller palpable mass was identified in the left breast. Palpation also revealed a mobile left axillary lymph node measuring approximately 2.1 cm.

Bilateral mammography demonstrated two suspicious breast lesions (Figure 1). In the right breast, a large, well-circumscribed, high-density, rounded mass measuring approximately 4.5-5 cm in its greatest dimension was identified. In the left breast, a smaller irregular high-density mass with partially indistinct margins, measuring approximately 3-3.5 cm, was observed in the upper inner quadrant. 

**Figure 1:**
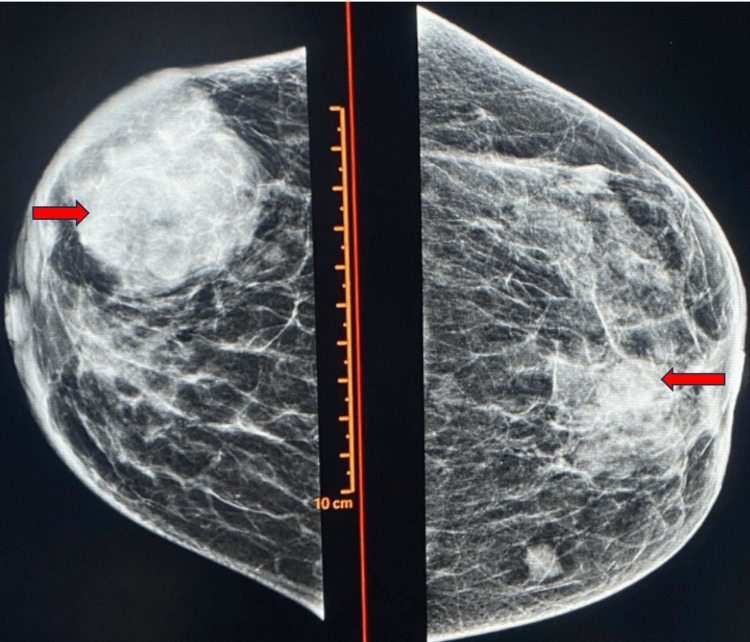
Bilateral mammography demonstrating a large right breast mass and a smaller left breast mass (red arrows).

A contrast-enhanced chest CT scan further demonstrated a right breast mass measuring approximately 24 × 21 mm (Figure 2). Although the disease was clinically bilateral, the available CT image is centered on the right-sided lesion. The discrepancy in tumor size between mammographic and CT measurements likely reflects differences in imaging modality and slice selection. 

**Figure 2:**
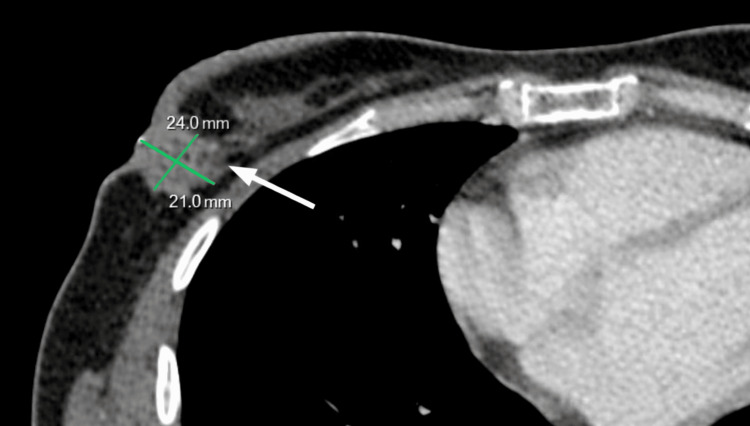
Contrast-enhanced axial CT scan of the chest showing a right breast mass (white arrow), measuring approximately 24 × 21 mm. Although the disease was clinically bilateral, the available image is centered on the right-sided lesion.

A core needle biopsy was subsequently performed. Histopathological examination revealed a malignant tumor arranged in solid sheets and trabecular patterns, composed of medium-to-large atypical cells. The tumor cells exhibited round-to-vesicular, hyperchromatic nuclei with irregular contours, fine chromatin, and inconspicuous nucleoli, associated with abundant eosinophilic cytoplasm. The tumor stroma was fibrous, and foci of tumor necrosis were identified (Figure 3). 

**Figure 3:**
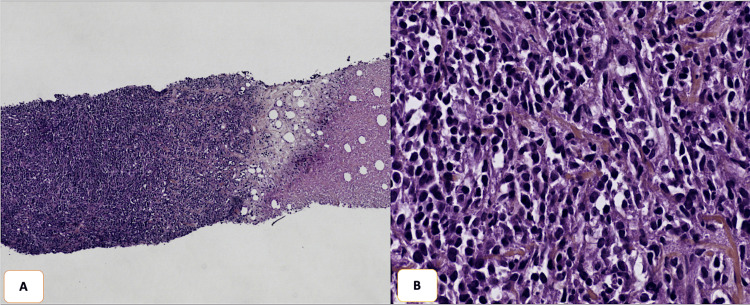
(A) Core biopsy showing a tumor proliferation arranged in diffuse sheets, with areas of tumor necrosis (H&E, ×5). (B) High-power magnification showing sheets of immature cells with fine nuclear chromatin (H&E, ×40).

Immunohistochemistry (IHC) analysis demonstrated strong expression of hematolymphoid and myeloid markers, including CD45 (leukocyte common antigen (LCA)), lysozyme, CD4, CD43, CD33, and CD68, with heterogeneous positivity for myeloperoxidase (MPO) and heterogeneous expression of CD117 (c-KIT). CD163 highlighted rare tumor cells. The neoplastic cells were negative for CD34, TdT, CD3, CD20, CD19, and CD10. Notably, PAX5 showed nuclear positivity in tumor cells, representing an aberrant expression, and CD79a was also expressed. In situ hybridization for immunoglobulin light chains demonstrated no κ/λ restriction (Figure 4 and Table 1). 

**Figure 4:**
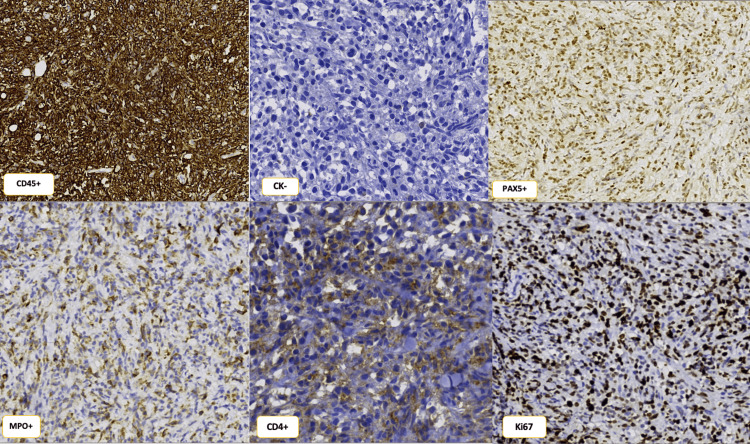
Immunohistochemical staining showing positive tumor cell labeling for CD45, PAX5, myeloperoxidase (MPO), and CD4. Cytokeratin (CK) is negative. The Ki-67 labeling index is approximately 60% (×20).

The detailed IHC profile is summarized in Table 1.

**Table 1:** Immunohistochemical profile of the breast tumor. LCA: leukocyte common antigen, MPO: myeloperoxidase, MS: myeloid sarcoma, ISH: in situ hybridization.

Marker	Result	Interpretation
CD45 (LCA)	Positive	Hematolymphoid origin
MPO	Positive (heterogeneous)	Myeloid differentiation
Lysozyme	Positive	Myeloid/monocytic lineage
CD33	Positive	Myeloid lineage
CD43	Positive	Frequently expressed in MS
CD117 (c-KIT)	Positive (heterogeneous)	Immature myeloid phenotype
CD68	Positive	Monocytic differentiation
CD163	Rare positive cells	Monocytic differentiation
CD4	Positive	Common in myelomonocytic MS
CD34	Negative	Does not exclude MS
TdT	Negative	Excludes lymphoblastic lymphoma
CD3	Negative	Excludes T-lineage
CD20	Negative	Excludes mature B-lineage
CD19	Negative	Excludes B-lineage
CD10	Negative	Excludes B-lineage
PAX5	Positive (aberrant)	Aberrant B-lineage marker expression
CD79a	Positive (aberrant)	Aberrant B-lineage marker expression
MUM1	Negative	Not supportive of plasma cell lineage
Cyclin D1	Negative	Excludes mantle cell lymphoma
κ/λ ISH	No restriction	No B-cell clonality

Given the strong expression of myeloid markers and the absence of convincing B-cell lineage differentiation despite PAX5 positivity, a primary lymphoid neoplasm was considered unlikely.

Molecular studies performed on the tumor tissue identified a KMT2A::MLLT10 fusion, confirming the myeloid nature of the neoplasm. Peripheral blood examination and bone marrow biopsy showed no evidence of circulating blasts or marrow involvement at the time of diagnosis. Based on the integration of morphological features, IHC profile, including aberrant expression of B-cell-lineage-associated markers, and molecular genetic findings, a diagnosis of primary (aleukemic) MS of the breast was established. The patient was subsequently referred to the hematology department, and systemic chemotherapy was initiated.

## Discussion

MS is an extramedullary tumor of immature myeloid cells that effaces normal tissue architecture and may arise at virtually any anatomic site. Contemporary classifications regard MS as the tissue counterpart of AML and support AML-directed management, with diagnosis based on an integrated framework combining morphology, immunophenotype, cytogenetics, and genomics [2-5,7,8]. Although MS commonly occurs in the setting of known AML, it may also present as an isolated, aleukemic mass without marrow or peripheral blood involvement at diagnosis. This subset is clinically important because the risk of subsequent systemic evolution remains substantial when therapy is limited to local approaches [6,9-11]; initial marrow negativity should therefore be regarded as a staging descriptor rather than an indicator of indolent biology.

Primary breast involvement is exceptionally uncommon and constitutes a diagnostic challenge, as clinical and radiologic findings frequently mimic carcinoma or lymphoma. Reported breast cases describe nonspecific imaging features (irregular enhancing masses, occasionally multifocal or bilateral), and a definitive diagnosis is typically established only after tissue evaluation with an appropriate IHC panel [12-14]. MS remains prone to misclassification even outside the breast, with recent reviews and series documenting clinically meaningful diagnostic error rates, most often lymphoma, of approximately 25%-47%, underscoring the need for systematic clinicopathologic correlation and early hematopathology input [15]. Extramedullary disease is uncommon in AML overall (granulocytic sarcoma/MS reported in ~1.4% of patients, and ~1.1% when AML is pooled with high-risk MDS) [11], and breast involvement is rarer still (~0.12% of AML diagnoses) [12], explaining how readily the entity is overlooked in standard breast oncology algorithms.

Histologically, MS shows a diffuse infiltrate of blasts with variable cytoplasm and nucleoli, sometimes with myelomonocytic features that can simulate high-grade carcinoma, lymphoma, or reactive inflammatory conditions on small biopsies. IHC is therefore pivotal and is best interpreted as a pattern rather than a single-marker result: strong myeloperoxidase (MPO) supports granulocytic differentiation, CD68 supports monocytic/histiocytic differentiation, CD43 is commonly expressed, and CD117 and CD34 vary with maturation stage and blast phenotype [7,15]. Because aberrant expression of nonmyeloid markers can occur and contribute to misdiagnosis, broad panels incorporating epithelial markers (cytokeratins), lymphoid markers, and multiple myeloid markers are recommended for any blastic, undifferentiated breast lesion [7,15]. In the present case, CD45 positivity with MPO and CD68 (and supportive lysozyme/CD33 with variable CD117/CD34) was highly consistent with MS once epithelial differentiation was excluded and mammary parenchyma was effaced.

The differential diagnosis is broad. Diffuse large B-cell lymphoma is a frequent pitfall given the discohesive "blastic" infiltrate, CD45 positivity, and the rare aberrant B-lineage expression of MS; integration of myeloid markers (MPO, lysozyme, CD33, CD117, CD68/CD163) with B- and T-cell markers is therefore essential [7,15]. Poorly differentiated or triple-negative breast carcinoma is another major mimic, where imaging may strongly favor carcinoma and negative broad cytokeratin staining supports exclusion of carcinoma and redirection toward hematolymphoid lineages [12-14]. Metastatic melanoma, plasma cell neoplasms, and histiocytic/dendritic cell tumors are additional considerations, particularly when CD68 is prominent, and demonstration of definitive myeloid differentiation (especially MPO positivity with concordant myeloid antigens) helps avoid misclassification [7,15]. Although PAX5 and CD79a are classically B-lineage markers, aberrant expression has been described in MS, especially in immature cases; their positivity should not preclude MS when the overall immunophenotype supports myeloid differentiation [5,7].

Cytogenetic and molecular studies further strengthen diagnostic confidence and inform risk stratification. Conventional karyotyping and FISH can detect recurrent AML-associated abnormalities (e.g., t(8;21), inv(16), and KMT2A rearrangements), supporting diagnosis and refining prognostic assessment [7,8]. Next-generation sequencing is increasingly relevant because it can identify AML-type and potentially actionable mutations [8]; profiling the breast specimen itself can add diagnostic precision and improve clinical decision-making in isolated breast MS [7,8].

Once MS is confirmed, a complete hematologic evaluation is indicated even in the absence of circulating blasts or cytopenias. Bone marrow aspirate/biopsy establishes whether disease is truly isolated and provides baseline staging, while cytogenetic and molecular testing may reveal AML-type alterations that influence prognosis and follow-up [2-6,8]. Therapeutically, evidence supports treating isolated MS as an AML-equivalent condition requiring AML-type systemic chemotherapy, as local therapy alone is associated with higher rates of progression to overt AML and inferior outcomes [2-5,8,9]. In a classic analysis of isolated MS/chloroma, early antileukemic chemotherapy was associated with a lower subsequent AML rate (41% vs 71%) than delayed or purely local approaches [9], and a series of nonleukemic granulocytic sarcoma treated predominantly with AML-type chemotherapy reported a complete remission rate of 65%, with radiotherapy serving as an adjunct rather than stand-alone therapy [10]. In contrast to primary breast carcinoma, surgery has no established therapeutic role beyond diagnosis; mastectomy and axillary dissection are not indicated [8,9]. Local radiotherapy may be considered for symptom relief or local control in selected cases, while systemic AML-type therapy remains the cornerstone, and consolidation with allogeneic stem cell transplantation may be considered for high-risk features or inadequate response within AML-directed frameworks [8-10].

Close follow-up is essential, as isolated breast MS can precede systemic AML or relapse at extramedullary sites even when marrow remains negative. Surveillance should be coordinated with hematology and include serial complete blood counts and clinical assessment, with repeat marrow evaluation guided by symptoms, cytopenias, circulating blasts, or new lesions on examination/imaging [8,9]. Overall, this case underscores that breast MS can clinically and radiologically mimic carcinoma and histologically mimic lymphoma or other undifferentiated malignancies; early consideration of MS, systematic IHC paneling, and integrated cytogenetic/molecular profiling are key to accurate diagnosis and timely AML-equivalent therapy [2-6,8-10,12-15].

## Conclusions

Primary (aleukemic) MS of the breast is a rare entity that can closely mimic common breast malignancies and is frequently at risk of misdiagnosis. Accurate recognition depends on morphology supported by a myeloid-directed IHC panel (notably MPO and monocytic markers), followed by cytogenetic and molecular profiling of the breast lesion to establish AML-equivalent biology, refine risk, and identify potentially targetable alterations. Because MS is a systemic disease equivalent to AML, treatment should consist of AML-type systemic chemotherapy, with radiotherapy reserved for selected local-control indications; breast surgery has no curative role beyond diagnostic sampling. Early, genomics-informed management and multidisciplinary coordination are critical to avoid unnecessary surgery and optimize patient outcomes.

## References

[1] Dock G (1983). Chloroma and its relation to leukemia. Am J Med Sci.

[2] Ngu IW, Sinclair EC, Greenaway S, Greenberg ML (2001). Unusual presentation of granulocytic sarcoma in the breast: a case report and review of the literature. Diagn Cytopathol.

[3] Brunning RD, Matutes E, and Flandrin G (2001). Myeloid sarcoma. Pathology and Genetics of Tumours of Haematopoietic and Lymphoid Tissues.

[4] Fitoz S, Atasoy C, and Yavuz K (2002). Granulocytic sarcoma. Cranial and breast involvement Clin Imaging.

[5] Pileri SA, Ascani S, Cox MC (2007). Myeloid sarcoma: clinico-pathologic, phenotypic and cytogenetic analysis of 92 adult patients. Leukemia.

[6] Pileri SA, Orazi A, Falini B (2017). Myeloid sarcoma. WHO Classification of Tumours of Haematopoietic and Lymphoid Tissues, 4th Edition.

[7] Khoury JD, Solary E, Abla O (2022). The 5th edition of the World Health Organization classification of Haematolymphoid tumours: myeloid and histiocytic/dendritic neoplasms. Leukemia.

[8] Arber DA, Orazi A, Hasserjian RP (2022). International Consensus Classification of Myeloid Neoplasms and Acute Leukemias: integrating morphologic, clinical, and genomic data. Blood.

[9] Döhner H, Wei AH, Appelbaum FR (2022). Diagnosis and management of AML in adults: 2022 recommendations from an international expert panel on behalf of the ELN. Blood.

[10] Almond LM, Charalampakis M, Ford SJ, Gourevitch D, Desai A (2017). Myeloid sarcoma: presentation, diagnosis, and treatment. Clin Lymphoma Myeloma Leuk.

[11] Patkowska E, Krzywdzinska A, Solarska I, Wojtas M, Prochorec-Sobieszek M (2025). Diagnostic approaches in myeloid sarcoma. Curr Issues Mol Biol.

[12] Diamantidis MD (2024). Myeloid sarcoma: novel advances regarding molecular pathogenesis, presentation and therapeutic options. J Clin Med.

[13] Naamo S, Naamo S, Sarker S, Vasconez M, Froicu M (2022). Breast manifestation of extramedullary myeloid sarcoma: a case report. Radiol Case Rep.

[14] Zhang Z, Chen Y, Zhang R, Liu M (2025). Primary breast myeloid sarcoma: a case report and literature review. Oncol Lett.

[15] Wu D, Li X, Tian X, Xu T, Ge Q, Luo S, Wang J (2025). Case report: difficulties in diagnosis of myeloid sarcoma of the breast by core needle biopsy. Front Med (Lausanne).

